# The Anti-Inflammatory Role of Bilirubin on “Two-Hit” Sepsis Animal Model

**DOI:** 10.3390/ijms21228650

**Published:** 2020-11-17

**Authors:** Duc Tin Tran, Yong Yeon Jeong, Jeong Min Kim, Hong Bum Bae, Sung Kuk Son, Sang Hyun Kwak

**Affiliations:** 1 Brain Korea 21 Project, Center for Creative Biomedical Scientists, Chonnam National University, Gwangju 61469, Korea; tintran.biomedical@gmail.com; 2 Department of Radiology, Chonnam National University Hwasun Hospital, Chonnam National University Medical School, Gwangju 61469, Korea; yjeong@jnu.ac.kr; 3 Department of Anesthesiology and Pain Medicine, Chonnam National University Medical School & Hospital, Gwangju 61469, Korea; tca77@chonnam.ac.kr (J.M.K.); nextphil2@jnu.ac.kr (H.B.B.); sonsungkuk123@gmail.com (S.K.S.)

**Keywords:** bilirubin, lipopolysaccharide, sepsis, anti-inflammation, cecal ligation and puncture

## Abstract

Introduction: Bilirubin is a product of the heme catabolism pathway, and it is excreted in bile and removed from the body through the urine. Bilirubin has potent antioxidant properties but also plays a role in anti-inflammation by protecting the body against endotoxin-induced lung inflammation, down-regulating the expression of adhesion molecules, and inhibiting the infiltration of inflammatory cells. Thus, bilirubin is a promising agent that could use in inflammation disease treatment. The application of bilirubin on the “two-hit” sepsis animal model has been, to date, unknown. Methods: we used lipopolysaccharide to induce initial insults in C57BL/6 mice. After 24 h, mice underwent cecal ligation and puncture to induce the “two-hit” sepsis model. Next, mice were administered 30 mg/kg bilirubin and we observed an improvement. Results: We observed that bilirubin inhibited the expression of pro-inflammatory cytokines, while the levels of anti-inflammatory cytokines were significantly augmented in the lung. Bilirubin improved the survival rate in the sepsis model. Furthermore, we suggest that bilirubin can modulate the accumulation of T-regulatory cells and myeloid-derived suppressor cells. Notably, bilirubin suppressed the activation and functions of T-cells. Conclusions: These results clarified that bilirubin might improve tissue injury in sepsis through anti-inflammatory mechanisms.

## 1. Introduction

Sepsis is the dysregulation of host responses to infection, which is associated with the dysfunction of single or multiple organs [1,2]. Pathogen-associated molecular patterns (peptidoglycan, lipoproteins, lipopolysaccharides, CpG-containing DNA, and flagellin) and damage-associated molecular patterns (high-mobility group box 1 and histones) are recognized by pattern recognition receptors on the surface of macrophages, neutrophils, dendritic cells, and lymphocytes. The presence of these may trigger the activation of intracellular signal pathways leading to systemic inflammatory response syndrome (SIRS) [1,3]. According to a previous study, SIRS involves the activity of complex intrinsic mediators during an acute phase reaction [4,5]. Early SIRS reactions are stimulated by initial microorganism insults, creating a so-called cytokine storm, recognized by the secretion of inflammatory mediators such as tumor necrosis factor-alpha (TNF-α), interleukin (IL)-6, and interferon-gamma (IFN-γ) [6]. In contrast to SIRS, compensatory anti-inflammatory response syndrome (CARS) supplies essential responses to reverse these processes. CARS terminate inflammatory responses and restore homeostasis by reducing antigen-presenting receptors on monocytes and increasing lymphocyte apoptosis, as well as activating the immunosuppressive cytokines IL-10 and transforming growth factor-β (TGF-β) [7]. However, uncontrolled overproduction of SIRS is aggravated in patients with sepsis, leading to early organ failures [1,5].

In the mid-1980s, European researchers indicated that sepsis was one of the significant factors leading to organ failure, with approximately 42% mortality of organ-failure patients diagnosed with sepsis. The lung is found to be the predominant dysfunctioning organ, found in almost all sepsis patients (approximately 74.9%) and with a high mortality (>50%) [8,9]. Organ failure was detected after the patient had suffered blunt trauma that was not identified at the site of infection [10]. On the other hand, a previous study also reported that, overwhelmingly, SIRS-induced infection and non-infection, and the outcome of uncontrolled sepsis (principally intra-abdominal infections) are the causes that trigger organ failure [11]. These factors cause exaggeration of the innate immune and inflammatory response, which releases free radicals, causing cellular perfusion failure, and increasing the production of reactive oxygen species and reactive nitrogen species [4,5]. This leads to the recruitment of large numbers of immune cells (macrophage, neutrophil, and lymphocyte) in the blood and organs, contributing to organ dysfunction [12]. In the clinical setting, the dysfunctional and initial extremely robust immune and inflammation response may cause fulminant death [11,13].

Bilirubin is an end product of heme catabolism and is synthesized by the activation of heme oxygenase during the catalysis and breakdown of heme and the reduction of biliverdin [14]. Bilirubin is known as an antioxidant and cytoprotective compound that attenuates cellular injury and protects neurons against damage induced by oxidative stress in vivo [15]. It also up-regulates extracellular superoxide dismutase and decreases superoxide production to prevent endothelial cell damage in diabetes [16]. Many reports have demonstrated the beneficial role of bilirubin in reducing tissue injury induced by inflammatory response stimulation [17]. Bilirubin exhibits significant anti-inflammatory ability in endotoxemia, septicemia, and ischemia-reperfusion injury animal models via mechanisms such as inhibiting the expression of adhesion molecules, suppressing the infiltration of inflammatory cells, and reducing the levels of pro-inflammatory cytokines [18]. Moreover, bilirubin has been shown to combat lipopolysaccharide (LPS), a bacterial product that induces liver injury and cardiovascular collapse [14,17]. On the other hand, a previous study showed that the heme oxygenase-knockout mice model to inhibit bilirubin production might lead to increased critical organ injury and mortality when challenged with LPS [19]. Therefore, bilirubin is a promising agent for the treatment of sepsis.

Sepsis-induced organ failure can occur in one of two ways: (i) a one-hit model with initial severe insult, and (ii) a two-hit model with sequential amplifying insults [11,20]. In this study, we used the “two-hit” sepsis model. Firstly, the mice were administrated 5 mg/kg intra-peritoneally (i.p) single-dose LPS for the initial sepsis insult. A previous study suggested that 5 mg/kg LPS may trigger serious clinical signs, prolong the recovery time and but did not cause death in a mice model, compared to mice that received 1.25 mg/kg LPS [21]. Next, we used medium-grade cecal ligation and puncture (CLP) severity to create sub-acute infectious peritonitis and amplify the inflammation response, leading to sepsis and fulminant death in the mice model. Finally, we injected the single-dose bilirubin in the “two-hit” sepsis mice model. The aim of the study was to investigate the effect of bilirubin on the sepsis model with a critical injury.

## 2. Results

### 2.1. Bilirubin Improves Survival in the “Two-Hit” Model

The mortality rate significantly increased in the “two-hit” group over time, approximately 70%, 50%, and 30% of mice (*n* = 10) surviving at 48 h, 76 h, 5 days post-CLP, respectively, with all mice dying 8 days after surgery. About 70% of mice in the CLP group had survived at 2 days after CLP, and no additional deaths were observed from day 2 to the end of day 14. There was no appearance of mortality when mice were challenged with LPS alone.

**Figure 1:**
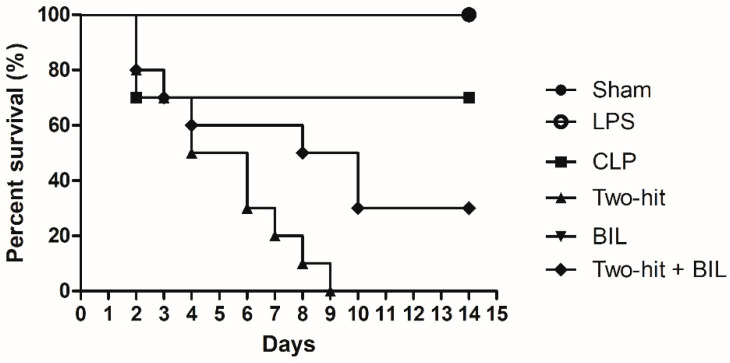
Bilirubin improves survival in the “two-hit” sepsis model. Mice were administered i.p. LPS at 5 mg/kg body weight on day 0. After 24 h, the mice were challenged with cecal ligation and puncture (CLP: 25-gauge needle and double-puncture). Next, the mice were administered bilirubin at 30 mg/kg body weight. The mice were observed for 15 days after LPS or saline was injected (*n* = 10 mice per group) and analyzed by Kaplan–Meier plots with log-rank tests. *p* < 0.001 two-hit group versus sham, LPS and BIL group. *p* < 0.01 two-hit group *versus CLP group. p* < 0.001 *two-hit + BIL group versus BIL group.* *p* = 0.040 in the “Two-hit” group versus two-hit + BIL group. LPS: lipopolysaccharide, CLP: cecal ligation and puncture, BIL: bilirubin.

To demonstrate the function of bilirubin in improving sepsis, 30 mg/kg body weight bilirubin was injected in mice in the “two-hit” model. We observed 20% of mortality mice in the “two-hit” + BIL groups compared with the BIL group within 24 h post-CLP surgery (day 2). The survival rate significantly improved in the “two-hit” + BIL group on day 8, and a 30% of survival rate was maintained after 14 days of monitoring compared to the “two-hit” group (Figure 1). From these observations, we demonstrated that bilirubin had a positive effect on the sepsis model.

### 2.2. The Effect of Bilirubin on Pro- and Anti-Inflammatory Cytokine Expression on the “Two-Hit” Model

To evaluate the level of pro- and anti-inflammatory cytokine expression in a tissue, we harvested the lung at 24 h post-CLP. The levels of pro-inflammatory cytokines in the lung tissues highly increased in the LPS, CLP, and “two-hit” group compared to the sham group. In the “two-hit” group, the level of TNF-α, IL-6, and IFN-γ cytokines was remarkably elevated compared to the remaining groups, in which excessive cytokines expression contributed to the aggravation of organ damage. In contrast, the “two-hit” group showed significantly low levels of IL-10 compared to the LPS group; and TGF-β compared to the LPS, CLP, and sham group.

**Figure 2:**
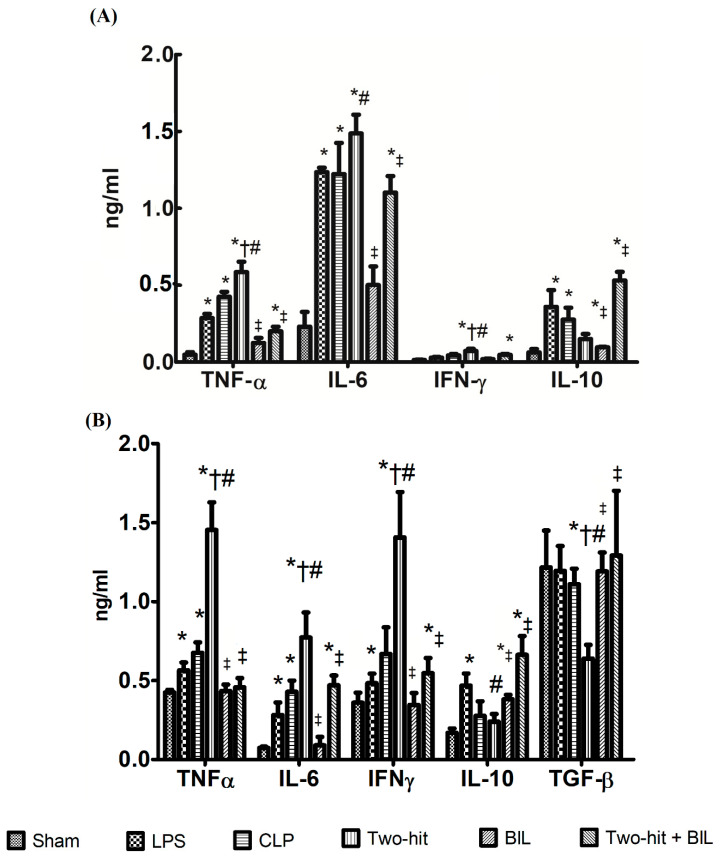
Bilirubin effects on the expression of pro- and anti-inflammatory cytokines in the (**A**) plasma; (**B**) lung of the “two-hit” sepsis model. Mice were administered i.p. LPS at 5 mg/kg body weight on day 0. After 24 h, the mice were challenged with cecal ligation and puncture (CLP: 25-gauge needle and double-puncture). Next, the mice were administered bilirubin at 30 mg/kg body weight. The levels of pro-inflammatory (TNF-α, IL-6, IFN-γ) and anti-inflammatory cytokines (IL-10 and TGF-β) were determined by ELISA after one day. Data are shown as mean ± SD (*n* = 5 mice per group) and analyzed by one-way ANOVA with Tukey–Krammer multiple comparison test using the SPSS program. * *p* < 0.05 versus sham. ^#^ *p* < 0.05 versus LPS. ^†^ *p* < 0.05 versus CLP. ^‡^ *p* < 0.05 versus “two-hit”. Tumor necrosis factor-alpha (TNF-α), interleukin (IL)-6, and interferon-gamma (IFN-γ), interleukin IL-10 and transforming growth factor-β (TGF-β). LPS: lipopolysaccharide, CLP: cecal ligation and puncture, BIL: bilirubin.

Next, we investigated whether bilirubin has anti-inflammatory capabilities and can protect tissue from the inflammatory response products. We measured the levels of pro-inflammatory (TNF-α, IL-6, and IFN-γ) and anti-inflammatory (IL-10 and TGF-β) cytokines in protein extracts from the lung of septic mice. We observed treatment with bilirubin on the “two-hit” model induced remarkable changes in pro-inflammatory cytokine expression. The expression of TNF-α, IL-6, and IFN-γ from the lung were attenuated compared to the “two-hit” group. Contrarily, the level of anti-inflammatory (IL-10, TGF-β) from the lung was significantly elevated in the “two-hit” + BIL group compared to “two-hit” (Figure 2B). On the other hand, we carried out similar evaluations about the level of pro- and anti-inflammatory cytokine expression in plasma and we realized that the outcomes were similar between lung and plasma (Figure 2A).

Briefly, the results showed that the overproduction of TNF-α, IL-6, and IFN-γ cytokines and low levels of anti-inflammatory cytokines is a factor and may lead to organ injury and early death in the “two-hit” group. The anti-inflammatory role of bilirubin may restrict organ dysfunction and protect cells from the toxic products of the inflammatory response and oxidative stress.

### 2.3. The Effect of Bilirubin on MAPKs Expression in the Lung in the “Two-Hit” Model

**Figure 3:**
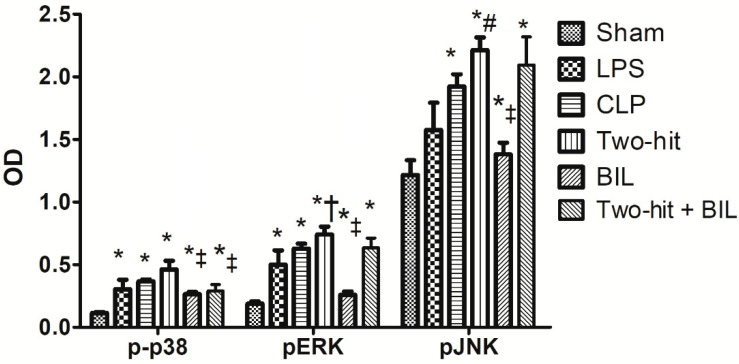
Bilirubin effects on the expression of MAPK kinase in the lung of the “two-hit” sepsis model. Mice were administered i.p. LPS at 5 mg/kg body weight on day 0. After 24 h, the mice were challenged with cecal ligation and puncture (CLP: 25-gauge needle and double-puncture). Next, the mice were administered bilirubin at 30 mg/kg body weight. The levels of MAPK kinase in the lung were determined by ELISA after one day. Data are shown as mean ± SD (*n* = 5 mice per group) and analyzed by one-way ANOVA with Tukey-Krammer multiple comparison test using the SPSS program. * *p* < 0.05 versus sham. ^#^ *p* < 0.05 versus LPS. ^†^ *p* < 0.05 versus CLP. ^‡^ *p* < 0.05 versus “two-hit”.

We measured ERK1/2, p38, and JNK expression in the lung at 24 h post-CLP. Activation of ERK1/2, p38, and JNK in the lung of the “two-hit” model significantly up-regulated compared to the sham, LPS, and CLP group. Next, we examined the effects of bilirubin on the “two-hit” models. As shown in Figure 3, we observed that bilirubin did not affect JNK and ERK1/2 phosphorylation, but result in attenuated p38 activation in the two-hit group-treated with bilirubin.

### 2.4. Bilirubin Enhances the Recruitment of Myeloid-Derived Suppressor Cells (MDSCs) and Suppresses the Activities and Functions of T Cells in Blood in the “Two-Hit” Model

**Figure 4:**
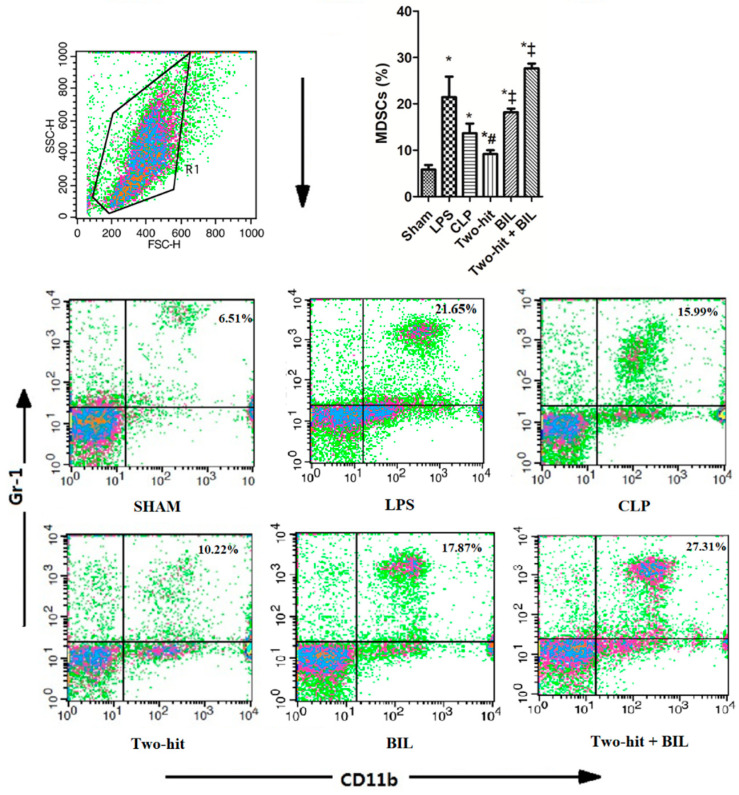
Bilirubin affects the recruitment of MDSC in the blood in the “two-hit” sepsis model. Mice were administered i.p. LPS at 5 mg/kg body weight on day 0. After 24 h, the mice were challenged with cecal ligation and puncture (CLP: 25-gauge needle and double-puncture). Next, the mice were administered bilirubin at 30 mg/kg body weight. The mice were sacrificed 24 h after CLP surgery to collect whole blood, and blood cells were stained to assess the percentages of MDSCs (CD11b^+^ Gr-1^+^). Data are shown as mean ± SD (*n* = 5 mice per group) and analyzed by one-way ANOVA with Tukey–Krammer multiple comparisons test using the SPSS program. * *p* < 0.05 versus sham. ^#^ *p* < 0.05 versus LPS. ^‡^ *p* < 0.05 versus “two-hit”.

**Figure 5:**
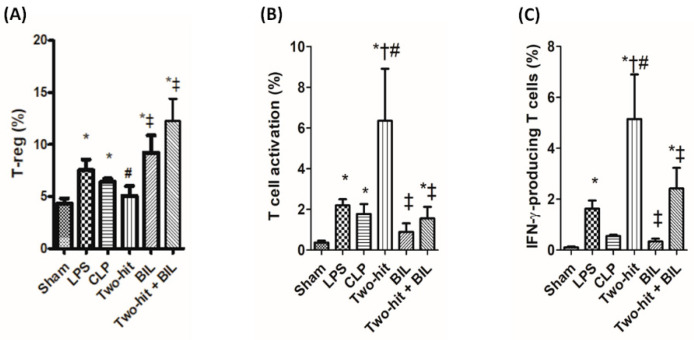
Bilirubin affects the recruitment of T-reg and the functions of T-cells in the blood in the “two-hit” sepsis model. Mice were administered i.p. LPS at 5 mg/kg body weight on day 0. After 24 h, the mice were challenged with cecal ligation and puncture (CLP: 25-gauge needle and double-puncture). Next, the mice were administered bilirubin at 30 mg/kg body weight. The mice were sacrificed 24 h after CLP surgery to collect whole blood, and blood cells were stained to assess the percentages of (**A**) T-reg (CD4^+^CD25^+^Foxp3^+^), (**B**) T-cell activation (CD3^+^CD28^+^), and (**C**) IFN-γ-producing cells in T-cells (CD3^+^IFN-γ^+^). Data are shown as mean ± SD (*n* = 5 mice per group) and analyzed by one-way ANOVA with Tukey–Krammer multiple comparisons test using the SPSS program. * *p* < 0.05 versus sham. ^#^ *p* <0.05 versus LPS. ^†^ *p* < 0.05 versus CLP. ^‡^ *p* < 0.05 versus “two-hit”.

Under the effect of pro-inflammatory cytokines (TNF-α, IL-6, and IFN-γ), MDSCs were activated. The accumulation of MDSCs in combination with T-reg cells leads to the suppression of immune cells (notably T-cell functions) and the modulation of macrophage product expression to prevent the harmful effects of the inflammatory response [22]. The immune cell population in blood was labeled with antibodies to detect MDSCs (CD11b^+^Gr-1^+^) and T-reg cells (CD4^+^CD25^+^Foxp3^+^). In the two hit group, we observed that MDSCs were significantly elevated, whereas T-reg did not show a difference compared to the sham group. Otherwise, the recruitment of MDSCs and T-reg cells in the “two-hit” group was lower than the LPS and CLP groups. The outcome showed that the disrupted equilibrium between SIRS and CARS in the two hit model might overwhelm the inflammatory response and augment the mortality of mice compared to LPS and CLP models. But when mice in the “two-hit” group were treated with bilirubin, the percentages of MDSCs and T-reg significantly accelerated, which contributed to the improvement of sepsis mice (Figure 4 and Figure 5A).

Next, we investigate the T cells activation in the blood of the “two-hit” group with and without treatment with bilirubin. The population of cells isolated from the blood at 24 h post-CLP was stained for a marker of T cells activation (CD3^+^CD28^+^) and IFN-γ- producing cells in T cells (CD3^+^IFN-γ^+^) [21,23]. We observed that T cell activation and IFN-γ-producing cells in the “two-hit” group were highly elevated compared to the sham, LPS, and CLP group. The over-activation of T cells is one of the reasons that may lead to aggravating tissue injury and early death in the “two-hit” model. But in the “two-hit” model with the presence of bilirubin, the percentage of T cell activation and IFN-γ-producing cells significantly reduced compared to the “two-hit” group (Figure 5B,C). These results confirmed the benefit of bilirubin in suppressing T cell activation and function, in turn contributing to decreased levels of major histocompatibility complex (MHC) class II molecules in monocytes/macrophages, and inhibiting immune response associated with the high recruitment of MDSCs and T-reg cells.

### 2.5. Bilirubin Improves the Lung Tissue Injury in the “Two-Hit” Model

**Figure 6:**
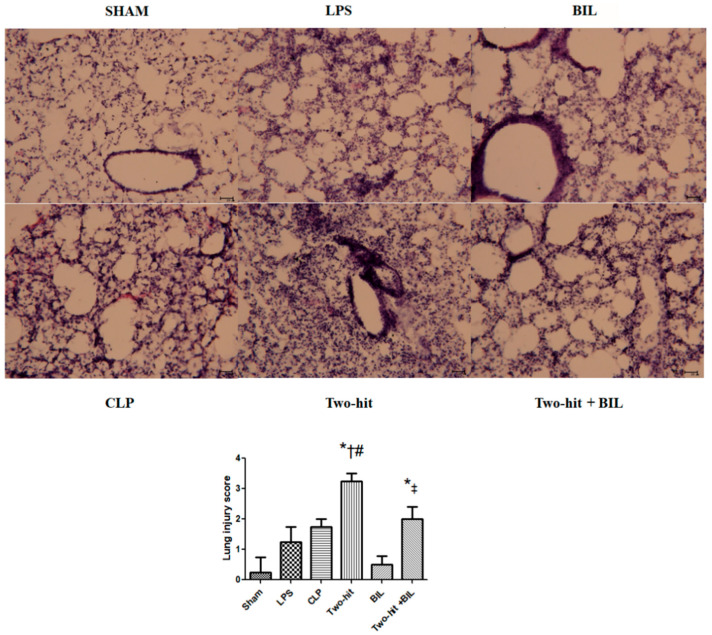
Lung tissue histology. Mice were administered i.p. LPS at 5 mg/kg body weight on day 0. After 24 h, the mice were challenged with cecal ligation and puncture (CLP: 25-gauge needle and double-puncture). Next, the mice were administered bilirubin at 30 mg/kg body weight. Histopathologic examination (H&E staining, original magnification X200) of the lung was performed after one day. The degree of lung injury was identified as described in the material and method part. Data are shown as mean ± SD (*n* = 5 mice per group) and analyzed by one-way ANOVA with Tukey–Krammer multiple comparisons test using the SPSS program. * *p* < 0.05 versus sham. ^#^ *p* < 0.05 versus LPS. ^#^ *p* < 0.05 versus LPS. ^†^ *p* < 0.05 versus CLP. ^‡^ *p* < 0.05 versus “two-hit”.

Under light microscopy, we found edema, hemorrhage, thickening of the alveolar wall, and infiltration of neutrophils in the lung of the LPS and CLP group, compared to the sham group, and more histological changes were observed in the “two-hit” group. There was no significant change between the sham group and bilirubin alone. However, the i.p administration of bilirubin reduced these pathologic changes compared to the two-hit group. Next, the severity degree of lung injury was ascertained semi-quantitatively. We suggested that the lung injury score significantly increased in the “two-hit” group than in the sham group and in the LPS and CLP groups, and was ameliorated in the bilirubin treatment (Figure 6).

## 3. Discussion

In recent years, bilirubin has been known as a toxic product of heme catabolism. The high concentrations of bilirubin may cause neurotoxicity and jaundice. However, Marc Jenniskens and et al. [19] suggested that mild hyperbilirubinemia could be lead to a beneficial “adapt and response” during sepsis in particular and critical illness in general. In this study, we observed that the serum bilirubin level increased in the two-hit + bilirubin group (approximately 0.22 mg/dL ± 0.04) compared with sham group (0.03 mg/dL ± 0.02) and two-hit group (0.13 mg/dL ± 0.03). These levels are still normal range (<0.5 mg/dL) [24]. Thus, the mild hyperbilirubinemia in the two-hit + bilirubin group may be a positive adaptive. Furthermore, many studies have demonstrated that because of its antioxidant ability, bilirubin exerts positive effects in protecting cells, notably neurons, against damage [15]. Knowledge regarding the physiological functions of bilirubin, in particular, its anti-inflammatory and immunomodulatory activities, has grown over time [18]. Given its various biological activities, bilirubin is a potential therapeutic agent for the treatment of sepsis. Based on recent studies regarding the anti-inflammatory and immunomodulatory abilities of bilirubin, we investigated the impact of bilirubin on the sepsis model. The “two-hit” model is a development of the acute sepsis model, but its more severe nature may lead to an early death. The benefits of bilirubin were observed through the modulation of pro- and anti-inflammatory cytokines in the lung. Besides, the accumulation of immune cells (such as MDSCs, T-reg) and the loss of T-cell functions in blood in the “two-hit” model demonstrate bilirubin’s benefits.

SIRS and CARS occur simultaneously during pathogen invasion. SIRS is characterized by the release of pro-inflammatory cytokines and immune system activation, whereas CARS terminates these processes and restores homeostasis through anti-inflammatory cytokines. When SIRS activation overwhelms CARS, it could lead to organ failure in the early phase and result in fulminant death [5]. The hallmark of sepsis-induced organ failure in the early phase is the amplification of pro-inflammatory cytokines, whereas the levels of anti-inflammatory cytokines were significantly attenuated. Similarly, we observed the over-expression of TNF-α, IL-6, and IFN-γ in the two-hit group and the low level of IL-10 and TGF-β, which stimulated an inflammatory response. This process resulted in high concentrations of ROS, nitric oxide, and free radicals, which aggravated organ damage and increased mortality in septic mice [12]. In contrast, bilirubin contributed to the up-regulation of anti-inflammatory cytokines and down-regulation of pro-inflammatory cytokines. Moreover, we found that bilirubin suppressed the phosphorylation of p-38 in the lungs of sepsis mice. The MAPKs, which consist of extracellular signal-regulated kinases 1 and 2 (ERK1/2), c-Jun N-terminal kinase (JNK), and p38, play a central role in modulating cellular signaling pathways, including proliferation, differentiation, apoptosis, and stress responses [25]. They also significantly contribute to the expression of inflammatory cytokines; the reduction of the signal pathway may lead to amelioration in sepsis mice. The level of pro-inflammatory cytokines and p38 expression in the treatment bilirubin group is still higher than the sham group but dramatically reduced compared to the two hit group. These showed that bilirubin could restrict the harmful effects on tissues during infection. The anti-inflammatory impact of bilirubin is the solution for treating sepsis, evidenced by the significant improvement in mortality after treatment with bilirubin.

High levels of TNF-α, IL-6, and IFN-γ are produced from T cells responses, and they contribute to the activation of the immune system against infection. Therefore, the loss of T cells function is associated with an impaired immune system, leading to a change from a hyper-inflammatory to a hypo-inflammatory state. However, the process is necessary for some patients with sepsis to avoid the over-expression of mediator products from the inflammatory response [21,26]. The loss of T cells function is an important means of restoring homeostasis during sepsis; bilirubin was demonstrated to have effects on T cells function in previous studies. Liu and et al. reported that bilirubin suppressed T cells proliferation and activation. T cell activation occurs via signals from the binding of antigen-specific T cell receptors (TCRs) with MHCs on antigen-presenting cells and signals from other interactions with costimulatory molecules. The loss of T cells function due to the effects of bilirubin leads to inhibits costimulatory molecules [27]. Our results similarly showed that T-cell activation and IFN-γ-producing cells in T cells significantly reduced with bilirubin treatment.

Furthermore, the suppression of T cells function involves the increase in regulatory T cells (Treg) and MDSCs [28]. The production of IFNγ, TLR ligands, TNF-α, PGE2, IL-1β, and IL-6 from T cells or bacterial infection may follow the accumulation MDSCs associated with signal transducer and activator of transcription signaling pathways [22]. The activation of MDSCs, led to the high expression of ROS, iNOS, arginase, and anti-inflammatory cytokines (IL-10 and TGF- β), contributes to the killing of bacteria and deactivation of inflammatory immune cells [29]. On the other hand, previous studies have reported that bilirubin promoted T-reg cells activity in vitro and generated T-reg cells in the mice model after administrating bilirubin, which was the supporting evidence for controlled inflammation response [30,31]. Moreover, the high number of MDSCs supports the stimulation of Tregs in the presence of IFN-γ and IL-10 [32]. Additionally, bilirubin showed the capacity to attenuate MHC class II expression in monocytes/macrophages, which led to decreased antigen presentation to T-cells [27,30]. The expression of inflammatory macrophages and the production of toxic substances from inflammatory responses attenuated in the presence of bilirubin [33]. Altogether, these observations support that inflammation and immune responses were reduced, which is a necessary condition for the treatment of sepsis.

Overall, our study provided valuable insight into the benefits of bilirubin on the sepsis model with critical injury, but these models still exist given limitations. The difference between mice strain, gender, supportive care method, and the effects of the environment may impact study outcomes. Moreover, the amelioration of the bilirubin-treated sepsis mice model is not representative of the improvement in sepsis patients. However, research using the mice model will be helpful for the development of new therapies. Our data helped to clarify a part of the beneficial effects of bilirubin, which may improve the mortality rate in sepsis through anti-inflammatory mechanisms. With these first advantages, bilirubin can be considered to further investigate the amelioration of organ failure in the sepsis model in the future.

## 4. Materials and Methods

### 4.1. Reagents

Antibodies against cell surface marker and detect intracellular pathways were purchased from BD Pharmingen (San Jose, CA, USA): CD11b-FITC, Gr-1-APC, FITC-CD3, PE-CD28, FITC-CD25, PE-Foxp3, APC-CD4, APC-IFN-γ, purified anti-CD16/CD32 (mouse BD Fc Block^TM^, BD Biosciences, Franklin Lakes, New Jersey, USA).

### 4.2. Preparation of Bilirubin

Bilirubin was purchased from Tokyo chemical industry Co., Ltd. (Tokyo, Japan), solubilized in 0.2 N sodium hydroxide and adjusted with 0.5 M hydrochloride acid to a pH of 7.4–7.6, as in a previous study [34]. The final concentration of bilirubin was diluted in saline solution; and stored –20 °C until use, preventing exposure to the light. All manipulations were carried out on a clean bench.

### 4.3. Animal

Six-week-old male C57BL/6 mice [21,35], with an average weight of 16–18 g were purchased from Dae Han Bio Link Co., Ltd. (Chungbuk, Korea). All mice were cared for in stable conditions and a specific pathogen-free environment. They were acclimated to the new environment for 1 week with an average weight 20–22 g prior. Next, the mice were divided into 6 other groups to make the model. All experiments were performed in accordance with our University Institutional Animal Care and Use Committees (CNU IACUC-H-2016-27).

### 4.4. Mice Models

#### 4.4.1. Sham Model and LPS Model

First of all, mice were administrated with intraperitoneal (i.p) 1 mL saline. After 24 h, the mice were anesthetized by isoflurane inhalation and maintained during surgery. Next, mice were opened for surgery, but their cecum was neither ligated nor punctured for the sham model. The mice were administered i.p LPS *055:B5 E. coli* (Sigma, St. Louis, MO, USA) 5 mg/kg body weight for the LPS group. All models were monitored twice daily for 14 days or until mortality.

#### 4.4.2. One-Hit Sepsis Model (Cecal Ligation and Puncture; CLP Model)

Cecal ligation and puncture (CLP) were used to induce the sepsis model, as described previously. First of all, mice were anesthetized by isoflurane inhalation and maintained during surgery. Next, the distal one-third of the cecum was ligated with 3-0 sterile silk and punctured twice with a 25-gauge needle. Then, the cecum was gently squeezed to extrude a few feces before returning the cecum into the abdomen. The abdominal wall and skin were closed in two layers. 1 mL pre-warm lactated Ringers plus 5% dextrose (with 0.05 mg/kg buprenorphine) was administered intraperitoneally (i.p) mice after surgery. In CLP with a 25-gauge needle; mice suffered the acute inflammatory response state and did not develop chronic infection [36,37], so we used the method to create the CLP model. All models were monitored twice daily for 14 days or until mortality.

#### 4.4.3. “Two Hit” Sepsis Model (Two-Hit Model)

The mice have injected with i.p lipopolysaccharide (LPS) *055:B5 E. coli* (Sigma, St. Louis, MO, USA) 5 mg/kg body weight, and after 24 h were operated on with a medium-grade CLP severity with a 25-gauge needle surgery to induce sub-acute sepsis infectious peritonitis. The protocol for surgery followed the previous research [21,38]. All models were monitored twice daily for 14 days or until mortality.

#### 4.4.4. Bilirubin Model (BIL Model)

First of all, mice were administrated with i.p 1 mL saline. After 24 h, the mice were anesthetized by isoflurane inhalation and maintained during surgery. Then, the mice were opened abdominally for surgery, but their cecum was neither ligated nor punctured. Next, the mice were administered bilirubin at 30 mg/kg body weight, as in a previous study [14]. All models were monitored twice daily for 14 days or until mortality.

#### 4.4.5. “Two Hit” Sepsis Model Treated with Bilirubin (Two-Hit + BIL Model)

The mice were challenged with i.p lipopolysaccharide (LPS) *055:B5 E. coli* (Sigma, St. Louis, MO, USA) 5 mg/kg body weight, and after 24 h were operated on with a medium-grade CLP severity with a 25-gauge needle to induced sub-acute sepsis infectious peritonitis. Next, the mice were administered bilirubin at 30 mg/kg body weight [14]. All models were monitored twice daily for 14 days or until mortality.

### 4.5. Measuring the Levels of Pro and Anti-Inflammatory Cytokines, Mitogen-Activated Protein Kinase (MAPK) from Lung by ELISA

#### 4.5.1. Collecting Lung Tissue

Mice from each group were collected after 24 h post-CLP to investigate the development of the sepsis process. At the time of sacrifice, mice inhaled isoflurane from a vaporizer until respiration ceased and mortality. Next, the lung was harvested from the mice. Tissue samples were incubated with RIPA lysis buffer (iNtRon, Biotechnology, Gyeonggi-do, South Korea) containing protease inhibitor (Merk-millipore, Seoul, Korea) to homogenize at 4 °C [39,40]. Homogenates were centrifuged at 13,000 rpm for 20 min and supernatants were removed into a new tube, store −80 °C until use. The protein concentration of each sample was assayed using the micro BCA protein assay kit standardized to BSA, following the manufacturer’s protocol.

#### 4.5.2. The Levels of Pro and Anti-Inflammatory Cytokines of the Lung

The levels of pro-inflammatory cytokine (tumor necrosis factor-alpha (TNF-α), Interleukin (IL)-6, and Interferon-gamma (IFN-γ)), and immunosuppressive cytokine (IL-10 and transforming growth factor (TGF-β)) were measured by ELISA kits (R&D Systems, Minneapolis, MN, USA), according to manufacturer’s instructions.

#### 4.5.3. The Levels of Mitogen-Activated Protein Kinase (MAPK)

Quantification of phosphorylated protein kinases [pT180/Y182] p38 MAPK, or [pTh183/Tyr185] JNK, or ERK 1/2 [pTh202/Tyr204] was detected in protein extracts from supernatants of the lung using a Simple Step ELISA Kit (Abcam, Eugene, OR, USA); according to the manufacturer’s instructions.

### 4.6. Tissue Histology

The tissue was fixed by 10% formaldehyde solution and embedded in OCT compound. Next, 10 μm sections of tissues were stained with hematoxylin and eosin. A pathologist, unaware of the nature of the experiment, observed under a light microscope and analyzed the samples. The change of histological lung injury score was assessed based on the infiltration of neutrophils, alveolar hemorrhage, inflammation, thickening of the alveolar septum, alveolar structure, using a previously published scoring system [41], between 0 = no or minimal injury, 1 = mild injury, 2 = moderate injury, 3 = severe injury, and 4 = maximum injury.

### 4.7. Determine the Percentages of Immune Cells (MDSC, T-Regulation, and Macrophage Polarization) from Blood by Flow Cytometry

To collect whole blood, mice were subjected to deep anesthesia, and whole blood was obtained via direct cardiac puncture using a sterile syringe with a 25-gauge needle containing 5 U heparin [42]. Next, whole blood (approximately 0.6 to 0.9 mL) was centrifuged 500 *g* for 5 min at 4 °C, transfer the whitish buffy coat layer to a new tube [43]. Erythrocytes were lysed by RBC lysis buffer BioLegend (San Diego, CA, USA). The pellets from whole blood were suspended in FBS stain buffer (BD pharmingen, Franklin Lakes, New Jersey, USA), which were used for analyzing cell staining. The serum was used to measure the levels of pro and anti-inflammatory cytokines.

#### Flow Cytometry

Cells were labeled with antibodies in ice-cold FBS stain buffer about 1 h at a concentration suggested by the manufacture. Intracellular staining of IFN-γ was performed after fixation with BD Cytofix/cytoperm^TM^ (BD Pharmingen, Franklin Lakes, New Jersey, USA) and washing with BD Perm/Wash buffer (BD Pharmingen, Franklin Lakes, New Jersey, USA) following the manufacturer’s instructions. Cells harvested from whole blood were analyzed by using a FACS Caliber flow cytometer (BD Biosciences, Sparks, MD, USA).

### 4.8. Statistical Analysis

The survival rates were determined by the Kaplan–Meier method, and the significantly different mortality was assessed by the log-rank test. Data were expressed as mean ± standard deviations (SD) for each group. Statistical significance was considered as *p-*value <0.05, *p-*value was analyzed by ANOVA or the Tukey–Kramer multiple comparisons test via the SPSS program. All experiments were performed at least three times, from three independent experiments.
